# The impact of human resources for health on the health outcomes of Chinese people

**DOI:** 10.1186/s12913-022-08540-y

**Published:** 2022-09-29

**Authors:** Jingjing Cheng, Xianming Kuang, Linghuang Zeng

**Affiliations:** 1 School of Business Administration, Northeastern University, Shenyang, 110819 Liaoning China; 2 Center for Economic Research, China Institute for Reform and Development, Haikou, 570311 Hainan China; 3 Human Resources Department, The First Affiliated Hospital of Hainan Medical University, Haikou, 570102 Hainan China

**Keywords:** Human resources for health (HRH), Residents’ health status, Data envelopment analysis (DEA), Malmquist index, Tobit regression, C33, D61, I11, I15, J24

## Abstract

Human resources for health (HRH) is a cornerstone in the medical system. This paper combined data envelopment analysis (DEA) with Tobit regression analysis to evaluate the efficiency of health care services in China over the years between 2007 and 2019. Efficiency was first estimated by using DEA with the choice of inputs and outputs being specific to health care services and residents’ health status. Malmquist index model was selected for estimating the changes in total factor productivity of provinces and exploring whether their performance had improved over the years. Tobit regression model was then employed in which the efficiency score obtained from the DEA computations used as the dependent variable, and HRH was chosen as the independent variables. The results showed that all kinds of health personnel had a significantly positive impact on the efficiency, and more importantly, pharmacists played a critical role in affecting both the provincial and national efficiency. Therefore, the health sector should pay more attention to optimizing allocation of HRH and focusing on professional training of clinical pharmacists.

## Introduction

Along with the development of the Chinese economy, there has been an increasing demand for health care services. Human resources for health (HRH), referencing the Joint Learning Initiative and the 2006 World Health Organization (WHO) health report, were capable of providing basic medical services and widely recognized as a crucial determinant of health system performance and of health outcomes [1]. The WHO’s report identified that one of the most important reasons for inefficiency in health care systems involved incentive mechanisms for health service providers [2]. Although many researchers have indicated that HRH availability has improved in some countries, out-of-balance [3–5], insufficient, and limited HRH availability [6, 7] persists in many other countries. Countries at all levels of socioeconomic development face difficulties in the deployment and performance of their HRH [8]. Many countries were facing a massive burden from the COVID-19 pandemic, and the healthcare systems were close to collapse in the most affected regions because of deprivation of human [9]. Therefore, optimizing the allocation of HRH is a top priority for healthcare systems, and the government should adopt a long-term perspective.

A large number of studies document the HRH in improving access to services and health outcomes. HRH constituted the infrastructure of health-care systems and delivered public-health, clinical, and environmental services. Development and support of the HRH are essential for achieving better health [10]. Strengthening HRH systems resilience ensures better health, and lessens preventable morbidity and mortality among mothers, babies and young children [11]. HIV constitutes an enormous global health burden. HRH were associated with an increase in HIV-positive case identification and critical to control HIV epidemic [12]. Pharmacists have been deemed the health professional nearest to residents and therefore particularly qualified to provide convenient and efficient medical care for everyday diseases [13–15]. Furthermore, HRH are the main force involved in dealing with public health emergencies. How well the country handles the COVID-19 crisis depends largely on how it uses the health workforce effectively and wisely [16]. While many doctors and nurses were fighting the uphill battle in the frontline, community health workers were poised to play a pivotal role in fighting the COVID-19 crisis, they maintain existing healthcare services while surging their capacity, interrupted the virus, and shield the most vulnerable from socioeconomic shocks [17]. Pharmacists have also made a great contribution to community health, apart from ensuring the stable supply of key medicines, they were also charged with the responsibility of early detection and appropriate referral [18]. HRH has played a vital role in achieving health and building the resilience of communities and health systems to respond to disasters caused by natural hazards.

HRH can improve health system efficiency. HRH consumes a substantial share of healthcare resources and determines the efficiency and overall performance of health systems [19]. Efficiency and productivity of health systems could be improved through optimizing health resource allocation, particularly between HRH and hospital beds [20]. Development of frontline and middle-level HRH has been a key component of successive health sector development programmes and improved health system efficiency [21]. Health economists have put forward a theory to welfare economics that health maximization is a resource allocation goal [22, 23]. One of the most challenging tasks is managing the workforces aligning with the health needs of the residents, and ensuring effective use of human and non-human resources [24]. Countries with inadequate health resources need to focus on health service efficiency and hospital resource productivity [25]. HRH is a cornerstone of the medical system, and benchmarking the human resources situation seems a prerequisite to any informed discussion of health care and health in China. The impact of HRH on the efficiency of health care services will be analyzed so that the government can modify health policy to improve the overall efficiency of health system.

## Literature review

Healthcare efficiency reflects how many HRH and resources in health system are needed to meet the demand for healthcare services among the residents. Data Envelopment Analysis (DEA) is the dominant approach to assessing the efficiency. Domestic scholars have studied the regional efficiency differences in China’s medical system. DEA approach was used to evaluate the China’s regional hospital efficiency with undesirable output [26], and assess the efficiency of health resources allocation in the 31 provinces of China [27]. Ding [28] found that the proportion of high-class medical facilities was negatively associated with the efficiency of healthcare systems in China. Foreign scholars have also widely researched efficiency and its contributing factors in the medical system [29–31]. DEA approach was used to measure Greek hospital performance and identified the factors that influenced their efficiency [32]. A two-stage approach was used to analyze the cost efficiency of critical access hospitals in the U.S [33], and the production performance of hospital services in Canada [34]. DEA-Malmquist index approach was used to analyze of the efficiency levels of Long-term care for a subset of OECD countries [35], and assess the productive efficiency of the national healthcare system of the ASEAN region [36]. A number of studies have shown that DEA approaches are widely used to assess healthcare system efficiency. Table 1 briefly summarizes the input and output variables developed in previous empirical studies that monitored efficiency.

**Table 1:** Input and output variables used in previous studies

Authors	Inputs	Outputs
Hamidi [37]	Number of beds, doctors, nurses, and non-medical staff	Number of treated inpatients and outpatients
Lee, Chun et al. [38]	Number of beds, doctors, and nurses	Number of inpatient and outpatient visits
Afonso and St. Aubyn [39]	Doctors, nurses, acute care beds, and MRI	Life expectancy, infant mortality, and potential years of life lost
Chen, Wu et al. [40]	Number of doctors, nurses, and beds	Number of outpatient visits and inpatient cases
Asandului, Roman et al. [41]	Number of doctors, hospital beds, and public health expenditures as percentage of gross domestic product (GDP)	Life expectancy at birth, health adjusted life expectancy, and infant mortality rate
Yang [42]	Population into three groups by year	Doctors, hospital beds, and medical expenditures
Ng [43]	Number of doctors, nurses, pharmacists, other staff, and beds	Number of outpatient and inpatient cases
Kontodimopoulos, Nanos et al. [44]	Number of doctors, nurses, and beds	Outpatient visits, admissions, and preventive medical services

Tobit regression analysis is used to reveal the factors affecting the efficiency of healthcare systems. Some scholars have compared efficiency scores before and after Chinese healthcare reform, and have revealed that the average overall efficiency was fluctuantly increasing after healthcare reform. Government health expenditures were positively associated with the efficiency of the healthcare system, but the number of public hospital and social health expenditure effected the efficiency in a negative way [45, 46]. DEA-Tobit regression was employed to examine the factors influencing the resource configuration efficiency of care services for elderly in Shanghai [47], evaluate the operational efficiency and quality of tertiary hospitals in Taiwan [48], and check the efficiency of healthcare systems and their determinants in the OECD countries [49]. Kim [50] measured hospital performance over 9 years by using the DEA-Malmquist Index approach and examined the impact of market competition on hospital efficiency in Pennsylvania by applying the panel Tobit analysis. Referencing the few scholars who have studied the efficiency of health services for the production of health outcomes and understanding the impact of HRH on the efficiency of health care services are a vital topic for health policy makers, and more empirical studies should be needed.

## Materials and methods

### Data sources and description

Panel data consist of 31 provinces and cities in China between 2007 and 2019. Time series data are the data set that arranges every one according to the time, which are available at only national and not provincial level. The panel data are used to analyze the efficiency at the provincial level, and the time series data are used to analyze the efficiency in China in general. This study used the data acquired from the *China Health Statistical Yearbook* from 2008 to 2020, including the data from the 2007 to 2019, which were published in the Chinese language by the Chinese central government. In case of a statistical divergence, the *China Health Statistical Yearbook* in 2020 shall prevail.

#### Input and output variables

The variable selection was guided by previous research reference Table 1 and and depended on the availability of data routinely compiled by the National Health Commission. We focused on analyzing the efficiency of the provincial and national levels, respectively. Three different inputs were used as health care services for the production of health outcomes. These inputs included number of outpatient visits, inpatient visits and surgeries. The health outcomes that we focus on are the standard measures of maternal, infant, under-five mortality, and life expectancy, which have been incorporated as indicators of the United Nations Millennium Development Goals [51], and the goals of residents’ health status in the “healthy China 2030” plan.

There has been a progressive decrease in the maternal infant and under-five mortality every year, while the variables in DEA originally were restricted to be non-negative. This paper applied a monotone decreasing transformation by taking the reciprocal of the maternal, infant, under-five mortality and multiplying the reciprocal by 100 or 10, which allowed the output variables to be positive. Because we still lack provincial data regarding infant and under-five mortality, these two variables were replaced with “perinatal mortality” and “prevalence rates of underweight children under-five” in the provincial level, respectively.

#### Independent variables

We considered HRH allocation as a possible influential factor on efficiency. In the Tobit regression analysis, efficiency scores were used as dependent variables, and the independent variables included doctors, nurses, pharmacists, technicians (include test and radiology technicians), and trainees (interns, practice nurse, etc.), which were assumed to impact the efficiency of health care services. We focused on maintaining data consistency. Efficiency scores (dependent variables) were between 0 and 1. This paper used the proportion of each HRH category (independent variables) instead. Table 2 presents variable selection.

**Table 2:** Model variables

Input: medical service (I)	Output: residents’ health status (O)	Independent variables (*x*)
Outpatient visits (I_1_)	Infant mortality (O_1_)	Licensed (assistant) doctors (*x*_1_)
Inpatient visits (I_2_)	under-five mortality (O_2_)	Registered nurses (*x*_2_)
Number of surgeries (I_3_)	Maternal mortality (O_3_)	Pharmacists (*x*_3_)
	Life expectancy (O_4_)	Technicians (*x*_4_)
		Trainees (*x*_5_)

#### Correlation analysis of input and output variables

We analyzed time trends of input and output variables in China, between 2007 and 2019. Table 3 shows a correlation analysis of input and output variables. Obviously, there were strong positive correlations between the variables. The correlation coefficient ranged from 0.914 to 0.993, demonstrating that medical services had a positive impact on residents’ health status.

**Table 3:** Correlation analysis of input and output variables in the national level

Input-output correlation		Infant mortality	Under-five mortality	Maternal mortality	Life expectancy
Outpatient visits	Pearson correlation	.944^a^	.954^a^	.952^a^	.985^a^
	significance	0.000	0.000	0.000	0.000
Inpatient visits	Pearson correlation	.982^a^	.983^a^	.993^a^	.989^a^
	significance	0.000	0.000	0.000	0.000
Number of surgeries	Pearson correlation	.956^a^	.954^a^	.914^a^	.916^a^
	significance	0.000	0.000	0.000	0.000

^a^denote significance at 5% statistical level

### DEA-Malmquist model

This study applied the DEA-Malmquist approach to estimate the efficiency of health care services in China. The first aspect is to analyze the efficiency at the provincial level from both the static and dynamic perspectives by using the panel data, and the second aspect is to analyze the efficiency in China in general by using a time series data. The DEA was first proposed in 1978 by Charnes and Cooper [52], two famous American operational research experts, and has great advantages in dealing with multiple input and output problems.

In Model (1), there are *n* Decision Making Units (DMUs), each of which uses *m* inputs to produce *s* outputs. *DMU*_*j*_(*j* = 1, 2, …, *n*) represents the *j*th DMU, where *x*_*j*_ = (*x*_1*j*_, …, *x*_*mj*_)^*T*^ is the input and *y*_*j*_ = (*y*_1*j*_, *y*_2*j*_, …, *y*_*sj*_)^*T*^ is the output for each DMU. Moreover, *v* = (*v*_1_, *v*_2_, …, *v*_*m*_)^*T*^ is the weight of the input and *u* = (*u*_1_, *u*_2_, …, *u*_*s*_)^*T*^ is the weight of the output. At the same time, *v* ∈ *E*^*m*^, *u* ∈ *E*^*s*^ make the efficiency evaluation index of *DMU*_*j*_:

1 $${\theta}_j=\frac{u^T{Y}_j}{v^T{X}_j}=\frac{\sum \limits_{r=1}^s{u}_r{y}_{rj}}{\sum \limits_{i=1}^m{v}_i{x}_{ij}},\left(j=1,2,\dots, n\right)$$

According to the basic ratio-based DEA model, we can always pick the right weight vector to make *θ*_*j*_ ≤ 1. Assuming that *DMU*_*o*_(*o* ∈ {1, 2, …, *n*}) is not optimal in these *n* decision units, we find the right *u* and *v* to maximize *θ*_*o*_. This particular DMU performance can be measured by the classic Charnes, Cooper and Rhodes’(CCR) programming model:

2 $${\displaystyle \begin{array}{l}\mathit{\operatorname{Maximize}}\frac{\sum \limits_{r=1}^s{\mu}_r{y}_{ro}}{\sum \limits_{i=1}^m{v}_i{x}_{io}}={\theta}_o\\ {} subject\kern0.5em to\frac{\sum \limits_{r=1}^s{\mu}_r{y}_{rj}}{\sum \limits_{i=1}^m{v}_i{x}_{ij}}\le 1,j=1,2,\dots, n\\ {}\kern2.52em {u}_{\mathrm{r}},{v}_i\ge 0,\forall r,i\end{array}}$$

It is difficult to calculate an optimal solution in Model (2), so conversion to a linear form using the Charnes-Cooper [53] transformation is necessary. Let $t=1/\sum \limits_{i=1}^m{v}_i{x}_{io}$, *μ*_*r*_ = *tu*_*r*_, (*r* = 1, 2, …, *s*) and *ω*_*i*_ = *tv*_*i*_, (*i* = 1, 2, …, *m*). Then, the model becomes

3 $${\displaystyle \begin{array}{l}\mathit{\operatorname{Maximize}}\sum \limits_{r=1}^s{\mu}_r{y}_{ro}={\theta}_0\\ {} subject\kern0.5em to\;\sum \limits_{i=1}^m{\omega}_i{x}_{io}=1\\ {}\kern4em \sum \limits_{r=1}^s{\mu}_r{y}_{rj}-\sum \limits_{i=1}^m{\omega}_i{x}_{ij}\le 0,j=1,2,\dots, n\\ {}\kern4em {\mu}_r,{\omega}_i\ge 0,r=1,2,\dots, s;i=1,2,\dots, m.\end{array}}$$

The CCR model is based on the assumption that the return to scale is constant. However, the medical industry is characterized by variable returns to scale. Banker, Charnes et al. [54] extended the CCR model to account for variable returns to scale, which became known as the Banker, Charnes and Cooper’s (BCC) model:

4 $${\displaystyle \begin{array}{l}\mathit{\operatorname{Maximize}}\sum \limits_{r=1}^s{\mu}_r{y}_{ro}-{u}_0\\ {} subject\kern0.5em to\;\sum \limits_{i=1}^m{\omega}_i{x}_{io}=1\\ {}\kern4em \sum \limits_{r=1}^s{\mu}_r{y}_{rj}-\sum \limits_{i=1}^m{\omega}_i{x}_{ij}-{u}_o\le 0,j=1,2,\dots, n\\ {}\kern4em {\mu}_r,{\omega}_i\ge 0,r=1,2,\dots, s;i=1,2,\dots, m.\end{array}}$$

Malmquist index was selected for estimating the changes in total factor productivity of DMUs and exploring whether their performance had improved over the years, which was named by Caves, Christensen et al [55] Färe, Grosskopf et al. [56] combined the Malmquist index with DEA, which is expressed as:

5 $${M}_i^{t+1}\left({x}_{t+1},{y}_{t+1},{x}_t,{y}_t\right)={\left[\frac{D_i^t\left({x}_{t+1},{y}_{t+1}\right)}{D_i^t\left({x}_t,{y}_t\right)}\times \frac{D_i^{t+1}\left({x}_{t+1},{y}_{t+1}\right)}{D_i^{t+1}\left({x}_t,{y}_t\right)}\right]}^{\frac{1}{2}}$$

T indicates the base period and t + 1 is the following period. (x_t_, y_t_) and (x_t + 1_, y_t + 1_) denote the input-output vector of China’s health care in period t and t + 1, respectively. It is worth noting that if the calculated score is greater than 1, it indicates increased efficiency. Whereas the calculated score is less than 1, it refers to a decline in efficiency. The calculated score = 1 does not reflect any changes in efficiency. Malmquist index can also be divided into two sub types [57]: change in technical and technological efficiency. This can be expressed as follows:

6 $${M}_i^{t+1}\left({x}_{t+1},{y}_{t+1},{x}_t,{y}_t\right)=\left(\frac{D_i^{t+1}\left({x}_{t+1},{y}_{t+1}\right)}{D_i^t\left({x}_t,{y}_t\right)}\right).{\left[\frac{D_i^t\left({x}_{t+1},{y}_{t+1}\right)}{D_i^{t+1}\left({x}_{t+1},{y}_{t+1}\right)}\times \frac{D_i^t\left({x}_t,{y}_t\right)}{D_i^{t+1}\left({x}_t,{y}_t\right)}\right]}^{\frac{1}{2}}$$

Where $\frac{D_i^{t+1}\left({x}_{t+1},{y}_{t+1}\right)}{D_i^t\left({x}_t,{y}_t\right)}$ refers to the change in technical efficiency.

${\left[\frac{D_i^t\left({x}_{t+1},{y}_{t+1}\right)}{D_i^{t+1}\left({x}_{t+1},{y}_{t+1}\right)}\times \frac{D_i^t\left({x}_t,{y}_t\right)}{D_i^{t+1}\left({x}_t,{y}_t\right)}\right]}^{\frac{1}{2}}$ defines the change in technological efficiency.

DEA offers input and output oriented models. The purpose of this paper is to study the efficiency of health resource allocation. We prefer to maximize the health of the population through using the medical services at present, which means that the objective is to maximize output by utilizing the same amount of input. These significant concerns will require the utilization of an output-oriented model under the assumption of variable returns to scale (VRS).

### Tobit regression

Once we calculated the efficiency scores by using DEA-Malmquist approach, a censored variable was formed and we used the Tobit regression to further examine determinant efficiency factors. The Tobit regression was first proposed by Tobit and the standard form is as follows [58]:

7 $${\displaystyle \begin{array}{c}{y}^{\ast }=\alpha +\beta {x}_i+{\mu}_i\\ {}{y}^{\ast }={y}_i\kern0.24em if\kern0.5em {y}^{\ast }>0\\ {}{y}^{\ast }=0\kern0.36em if\kern0.5em {y}^{\ast}\le 0\end{array}}$$

Where *y*^∗^ is the potential dependent variable, *α* is the constant term, and *x*_*i*_ is the impact factor, *β* is the coefficient vector, and *μ*_*i*_ is the error term. The Tobit regression follows a normal distribution. In this study, we generally used the following form [59, 60]:

8 $${y}^{\ast }=\beta {x}_i+{\mu}_i,$$

For this study’s purpose, the Tobit regression equations were established as follows:

9 $${\displaystyle \begin{array}{c}{EFF}_j^1={\alpha}_1{x}_{1j}+{\alpha}_2{x}_{2j}+{\alpha}_3{x}_{3j}+{\alpha}_4{x}_{4j}+{\alpha}_5{x}_{5j}+{\mu}_j\\ {}{EFF}_t^2={\beta}_1{x}_{1t}+{\beta}_2{x}_{2t}+{\beta}_3{x}_{3t}+{\beta}_4{x}_{4t}+{\beta}_5{x}_{5t}+{\varepsilon}_t.\end{array}}$$

where ${EFF}_j^1$ represents the 2019 Chinese provincial efficiency scores, *j*(*j* = 1, …, 31) represents the 31 provinces, and ${EFF}_t^2$ indicates China’s national efficiency scores. While *t*(*t* = 1, …, 13) represents each year from 2007 to 2019, *x*_*ij*_, *x*_*it*_ indicates the impact factors, and *α*_*i*_, *β*_*i*_ represents the regression coefficient.

### Theil index

In 1967, Thiel proposed the Theil index, which was a measure of inequality based on the information theory [61]. It can be written as

10 $$T=\frac{1}{n}\sum \limits_{i=1}^n\frac{y_i}{\overline{y}}\log \left(\frac{y_i}{\overline{y}}\right),$$

Where *T* is the Thiel index, *y*_*i*_ represents the actual value of the observed indicator in region *i*, and $\overline{y}$ represents the average value of all regional observations. The smaller the Thiel index, the smaller the distribution difference will be.

While the results of the DEA and Malmquist index were obtained using DEAP 2.1 software, the results of the Tobit regression analysis were obtained using EViews 10 software and the results of the Theil index using R × 64 3.6.1 software.

## Results

### Static analysis of medical services efficiency at the provincial level

The results of the DEA estimations are presented in the Table 4 for the year 2007 and 2019. The provinces like Tianjin, Hainan and Tibet were on the frontier and thus considered as efficient provinces in both the two periods, Beijing, Qinghai and Ningxia achieved maximum efficiency only in 2019. The mean overall efficiency (crste) was increasing over time, from 0.355 in 2007 to 0.416 in 2019, which was still far away from the production possibility frontier and considered to be relatively inefficient. Likewise, the scale efficiency (scale) on average was also inefficient, which increased from 0.368 in 2007 to 0.427 in 2019. From the overall efficiency perspective, the provinces of full efficiency were consistent with the ones of full scale efficiency. In terms of the pure technical efficiency (vrste), the values were 0.946 in 2007 and 0.955 in 2019, indicating more efficient than overall efficiency (crste) and scale efficiency (scale). The 4 provinces that achieved maximum pure technical efficiency (vrste) included Tianjin, Shanghai, Hainan, Tibet in 2007. Four provinces were added with an efficiency value of 1 in 2019, namely Beijing, Jilin, Qinghai, Ningxia. In 2019, these provinces all showed a trend of decreasing returns to scale except for Beijing, Tianjin, Hainan, Tibet, Qinghai and Ningxia. (Table 4).

**Table 4:** Efficiency value of medical services in 31 provinces in 2007 and 2019

Province	2007				2019			
	crste	vrste	scale	rts	crste	vrste	scale	rts
Beijing	0.394	0.985	0.400	drs	1.000	1.000	1.000	–
Tianjin	1.000	1.000	1.000	–	1.000	1.000	1.000	–
Hebei	0.199	0.940	0.211	drs	0.155	0.935	0.165	drs
Shanxi	0.275	0.956	0.288	drs	0.381	0.950	0.401	drs
Inner Mongolia	0.367	0.939	0.391	drs	0.454	0.954	0.476	drs
Liaoning	0.285	0.954	0.299	drs	0.224	0.959	0.234	drs
Jilin	0.340	0.974	0.350	drs	0.789	1.000	0.789	drs
Heilongjiang	0.318	0.958	0.332	drs	0.328	0.968	0.339	drs
Shanghai	0.604	1.000	0.604	drs	0.853	1.000	0.853	drs
Jiangsu	0.185	0.946	0.196	drs	0.133	0.955	0.140	drs
Zhejiang	0.256	0.973	0.263	drs	0.245	0.968	0.253	drs
Anhui	0.261	0.946	0.276	drs	0.183	0.936	0.196	drs
Fujian	0.230	0.953	0.242	drs	0.279	0.945	0.295	drs
Jiangxi	0.316	0.914	0.345	drs	0.325	0.971	0.334	drs
Shandong	0.164	0.946	0.174	drs	0.100	0.953	0.105	drs
Henan	0.117	0.916	0.127	drs	0.094	0.929	0.102	drs
Hubei	0.216	0.922	0.234	drs	0.210	0.933	0.225	drs
Hunan	0.179	0.928	0.193	drs	0.207	0.931	0.223	drs
Guangdong	0.082	0.938	0.088	drs	0.084	0.953	0.088	drs
Guangxi	0.194	0.932	0.208	drs	0.174	0.936	0.186	drs
Hainan	1.000	1.000	1.000	–	1.000	1.000	1.000	–
Chongqing	0.371	0.972	0.382	drs	0.310	0.954	0.325	drs
Sichuan	0.102	0.911	0.112	drs	0.098	0.931	0.105	drs
Guizhou	0.287	0.891	0.323	drs	0.219	0.895	0.245	drs
Yunnan	0.138	0.863	0.160	drs	0.139	0.867	0.160	drs
Tibet	1.000	1.000	1.000	–	1.000	1.000	1.000	–
shaanxi	0.226	0.927	0.244	drs	0.289	0.937	0.309	drs
Gansu	0.233	0.909	0.256	drs	0.357	0.915	0.390	drs
Qinghai	0.824	0.965	0.853	drs	1.000	1.000	1.000	–
Ningxia	0.676	0.981	0.690	drs	1.000	1.000	1.000	–
Sinkiang	0.152	0.898	0.169	drs	0.275	0.918	0.299	drs
mean	0.355	0.946	0.368		0.416	0.955	0.427	

*crste* Overall efficiency, *vrste* Pure technical efficiency, *scale* Scale efficiency, *rts* Return to scale, *drs* Decreasing; *irs* Increasing

### Dynamic analysis of medical services efficiency at the provincial level

Table 5 reveals the Changes in China’s medical services efficiency between 2007 and 2019. The mean technical efficiency change (effch) value indicates a 0.7% increase, with the highest increase of 25.3% between 2015 and 2016, and 16.1% between 2009 and 2010. However, the mean technological change (techch) value of 0.932 indicates an average annual decrease of 6.8% in technological advances, with a larger regress of 47.2% between 2008 and 2009. which plays a major role in reducing total factor productivity change (tfpch). The tfpch has shown a similar productivity decline during the study period, with the largest regress of 51.7% between 2008 and 2009. On an annual basis, only in the periods of 2007 to 2008, 2009 to 2010, 2010 to 2011, 2014 to 2015, and 2017 to 2018 did the total factor productivity change (tfpch) of China’s medical services increase, while all other years showed a decline.

**Table 5:** Malmquist index of medical services between 2007 and 2019 in China

Year	effch	techch	pech	sech	tfpch
2007–2008	1.087	0.946	1.050	1.035	1.028
2008–2009	0.916	0.528	0.904	1.013	0.483
2009–2010	1.161	0.863	1.132	1.026	1.003
2010–2011	0.997	1.158	1.031	0.967	1.154
2011–2012	0.966	0.985	0.928	1.042	0.952
2012–2013	1.017	0.931	1.131	0.899	0.946
2013–2014	0.999	0.941	1.060	0.943	0.940
2014–2015	0.957	1.084	0.947	1.010	1.037
2015–2016	1.253	0.744	1.090	1.150	0.932
2016–2017	0.764	1.258	0.765	0.999	0.961
2017–2018	1.018	1.037	1.026	0.992	1.056
2018–2019	1.025	0.953	1.004	1.022	0.977
mean	1.007	0.932	1.000	1.006	0.938

effch, technical efficiency change; techch, technological change; pech, pure efficiency change; sech, scale efficiency change; tfpch, total factor productivity change

Table 6 indicates the Malmquist index of medical services in 31 provinces between 2007 and 2019. As can be seen, The mean technical efficiency change (effch) value of 1.007 suggests a paltry technical efficiency increase of 0.7%. Specifically, as manifested in the provinces results, technical efficiency change (effch) grew mildly by 8.1% in Beijing, 7.3% in Jilin and 5.4% in Sinkiang. While 61.3% of the provinces had a technical efficiency progress during 2007–2019, the rest of the provinces suffered a technical efficiency regress or had their stagnation. Comparably, the mean technological change (techch) value of 0.932 highlights that the technology of medical services across provinces shrinks slowly and that in turn affects the total output growth over time. The total factor productivity change (tfpch) on average also follows a similar declining pattern of 0.938, with Tianjin, Shanghai, Chongqing and Guizhou showing the largest decreases, reaching 13.4, 10.9, 9.2 and 8.9%, respectively. Notably, all the provinces had technological (techch) and total factor productivity change (tfpch) regress.

**Table 6:** Malmquist index of medical services in 31 provinces between 2007 and 2019

DMU	effch	techch	pech	sech	tfpch
Beijing	1.081	0.851	1.034	1.045	0.919
Tianjin	1.000	0.866	1.000	1.000	0.866
Hebei	0.979	0.969	1.029	0.952	0.949
Shanxi	1.030	0.947	1.029	1.001	0.975
Inner Mongolia	1.018	0.915	1.012	1.006	0.931
Liaoning	0.981	0.946	0.980	1.001	0.928
Jilin	1.073	0.890	1.083	0.991	0.954
Heilongjiang	1.002	0.937	1.002	1.000	0.939
Shanghai	1.029	0.865	1.000	1.029	0.891
Jiangsu	0.973	0.963	0.946	1.029	0.937
Zhejiang	0.996	0.943	0.947	1.051	0.940
Anhui	0.971	0.946	0.997	0.974	0.918
Fujian	1.016	0.957	1.020	0.996	0.973
Jiangxi	1.002	0.951	1.038	0.966	0.953
Shandong	0.960	0.956	0.937	1.024	0.918
Henan	0.982	0.949	0.954	1.030	0.933
Hubei	0.998	0.951	0.968	1.031	0.949
Hunan	1.013	0.930	1.007	1.005	0.942
Guangdong	1.002	0.955	0.990	1.012	0.957
Guangxi	0.989	0.956	0.991	0.998	0.946
Hainan	1.000	0.966	1.000	1.000	0.966
Chongqing	0.985	0.922	0.919	1.072	0.908
Sichuan	0.997	0.937	0.997	1.000	0.934
Guizhou	0.978	0.932	0.967	1.011	0.911
Yunnan	0.998	0.950	1.000	0.997	0.948
Tibet	1.000	0.918	1.000	1.000	0.918
shaanxi	1.021	0.942	1.044	0.978	0.961
Gansu	1.040	0.918	1.036	1.004	0.955
Qinghai	1.016	0.941	1.014	1.002	0.956
Ningxia	1.035	0.912	1.034	1.001	0.944
Sinkiang	1.054	0.930	1.050	1.004	0.980
mean	1.007	0.932	1.000	1.006	0.938

*DMU* Decision Making Unit, *effch* Technical efficiency change, *techch* Technological change, *pech* Pure efficiency changem *sech* Scale efficiency change, *tfpch* total factor productivity change

### The medical services efficiency in China at the national level

In the national level, each year was treated as a Decision Making Unit (DMU). There is a rule of thumb that the number of units should be at least twice the number of inputs and outputs in order to preserve discriminatory power [62–65]. Therefore, we employed the factor analysis to reduce the dimension of health outcomes variables:

11 $$y={0.992}^{\ast }{o}_1+{0.996}^{\ast }{o}_2+{0.993}^{\ast }{o}_3+{0.973}^{\ast }{o}_4$$

where *y* represents the health outcomes, *o*_1_, *o*_2_, *o*_3_ represent the positive change of infant, under-five and maternal mortality respectively, and *o*_4_ represents the life expectancy. Input variables were the same as the provincial analysis mentioned above, including number of outpatient visits, inpatient visits and surgeries.

Table 7 shows that the pure technical efficiency was obviously higher than the overall efficiency and scale efficiency. The overall efficiency, which were lower than 1 during 2008–2019, indicated a poor efficiency level. The scale efficiency shrank slowly and in turn affected the total output growth over time. The pure technical efficiency in the year including 2007, 2015, 2018 and 2019 was on the frontier and thus considered as efficient period.

**Table 7:** Efficiency value of medical services in China at the national level

Year	Overall efficiency	Pure technical efficiency	Scale efficiency	Return to scale
2007	1	1	1	–
2008	0.97	0.994	0.976	drs
2009	0.881	0.989	0.89	drs
2010	0.839	0.986	0.851	drs
2011	0.797	0.982	0.811	drs
2012	0.75	0.99	0.758	drs
2013	0.722	0.994	0.727	drs
2014	0.706	0.985	0.716	drs
2015	0.715	1	0.715	drs
2016	0.705	0.992	0.711	drs
2017	0.702	0.995	0.706	drs
2018	0.713	1	0.713	drs
2019	0.697	1	0.697	drs

*drs* Decreasing, *irs* Increasing

**Fig. 1:**
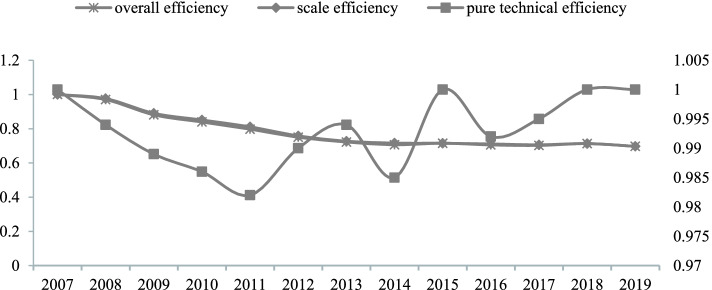
Medical services efficiency in China at the national level. Note: Pure technical efficiency were on the secondary axis

The efficiency scores are plotted in diagram (Fig. 1) from 2007 to 2019. As can be seen, the overall efficiency was varying in a way similar to the scale efficiency, and all showed a trend of decreasing. The pure technical efficiency appeared to fluctuate from 2011 to 2016, which is influenced by the healthy and medical departments management and technologies, and perhaps part of the reason lies with healthy public policies adjustment.

### The impact of HRH on the provincial efficiency

Table 8 shows the estimation results of the Tobit regression analysis from the provincial level. In this analysis, the overall efficiency (crste), pure technical efficiency (vrste) and scale efficiency (scale) were used as dependent variables respectively. The impact factors in the pure technical efficiency (vrste) model were slightly different. Except for technicians (*x*_4_), every other HRH category in the pure technical efficiency (vrste) model was statistically significant(*p* < 0.05) and positive, while only pharmacists(*x*_3_) and nurses(*x*_2_) were significant(*p* < 0.05) in the overall efficiency (crste) and scale efficiency (scale) model. When analyzing the correlation coefficient, the largest one was for pharmacists in all the three models, followed by doctors. More importantly, pharmacists were found to be 3 or 20 times more influential than doctors. Therefore, we concluded that the pharmacists have a generally significant impact on the provincial efficiency. The correlation coefficient of the nurses(*x*_2_) was positive in the pure technical efficiency (vrste) model, but it was negative in the overall efficiency (crste) and scale efficiency (scale) model. As mentioned above, the scale efficiency was still on the low side. A trend of decreasing returns to scale was showed in most of the provinces, which highlights that higher proportion of nurses was clearly associated with lower scale efficiency. Whereas, the insignificant coefficients recorded for technicians cannot be taken to conclude that they do not matter.

**Table 8:** The impact of HRH on the provincial efficiency

Variable		Crste model		Vrste model		Scale model	
		Coefficient	Prob.	Coefficient	Prob.	Coefficient	Prob.
doctors	*x*_1_	1.099	0.412	1.093	0.000	1.071	0.418
nurses	*x*_2_	−3.503	0.022	0.464	0.001	−3.413	0.024
pharmacists	*x*_3_	20.588	0.029	3.860	0.000	20.040	0.031
technicians	*x*_4_	8.046	0.589	1.998	0.131	8.049	0.585
trainees	*x*_5_	1.409	0.647	0.578	0.032	1.508	0.620

*crste* Overall efficiency, *vrste* Pure technical efficiency, *scale* Scale efficiency

### The impact of HRH on the National Efficiency

Table 9 shows the estimation results of the Tobit regression analysis for the national level efficiency. It can be seen that the impact factors on the national efficiency were slightly different from the ones on provincial efficiency. The technicians (*x*_4_) turned to be significant and had a negative impact on the efficiency. Doctors(*x*_1_) and nurses(*x*_2_) had significant(*p* < 0.05) and positive effects on the pure technical efficiency (vrste), while pharmacists(*x*_3_) and trainees(*x*_5_) were only statistically significant in the overall efficiency (crste) and scale efficiency (scale) model. In the overall efficiency (crste) model, the coefficient of pharmacists was the largest, which was consistent with results of the Tobit regression from the provincial level and was much higher than nurses. Moreover, we can noticed that the coefficient of technicians and trainees was negative, the absolute value of that numbers was still much higher than the coefficient of nurses.

**Table 9:** The impact of HRH on the national efficiency

Variable		Crste model		Vrste model		Scale model	
		Coefficient	Prob.	Coefficient	Prob.	Coefficient	Prob.
doctors	*x*_1_	0.129	0.889	2.096	0.000	−0.179	0.837
nurses	*x*_2_	0.935	0.044	1.265	0.000	0.684	0.119
pharmacists	*x*_3_	63.558	0.000	7.013	0.152	57.472	0.000
technicians	*x*_4_	−38.221	0.004	−12.399	0.015	−29.823	0.018
trainees	*x*_5_	−9.444	0.000	−0.200	0.726	−8.563	0.000

crste, overall efficiency; vrste, pure technical efficiency; scale, scale efficiency

### Results of Theil index

**Fig. 2:**
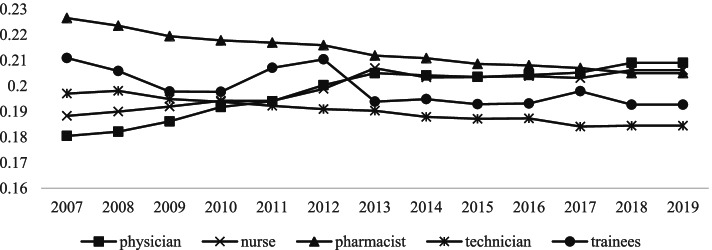
The Theil indexes of HRH distribution in China

Unequal allocation of HRH represents one of the major problems in the current medical service management in many countries, and analyzing the equity of HRH allocation is a key step to improve the health system efficiency. Many scholars have found that health resources are inefficient because of excess demand for healthcare and uneven distribution [66, 67]. The inequity of HRH distribution was found in urban community health service in China, especially in quality and geographic [68]. China’s health-equity challenges are truly daunting. The social determinants of health have become more inequitable, and concerns among the public have grown about fairness in health [69]. Medical service equity is a key factor for social fairness, but service delivery and health resource allocation in China are still inefficient [70]. Theil index is often used to estimate the fairness of the HRH distribution [71, 72]. For further discussion, we used the Theil index to measure inequality trends of HRH distribution among provinces. Figure 2 shows a daunting equity challenge in China. There is uneven distribution of HRH between provinces generally, and the most severe inequality was pharmacists. It is also worth noting that the inequality distribution of doctors and nurses remarkably increased, while that of technicians and trainees was the opposite. For further analyzing, if the inequity of HRH had the impact on the health care services efficiency, the Theil index came to the conclusion, similarly, that the pharmacists had greater impact on the efficiency, and that one of the important reasons was probably for the highest Theil index.

## Discussion

From the dynamic analysis results of medical services efficiency at the provincial level, the mean technical efficiency change (effch) value indicates a 0.7% increase, with the highest increase of 25.3% between 2015 and 2016, and 16.1% between 2009 and 2010, suggesting that the medical services system has integrated technical innovation. In 2009, the Communist Party of China (CPC) put forward the opinions on deepening the reform of the medical and health care system, which marked the beginning of a new health care reform. The comprehensive reform of county-level public hospitals has been practiced in 2015, marking the reform of public hospitals at the county level. This contributed significantly to the increase in total factor productivity change (tfpch). As manifested in the provinces results, the mean technological change (techch) value affects the total output growth over time. China’s efforts to improve medical services efficiency mainly arise from the the promotion of technical progress. In terms of the Tobit regression analysis resulting for the national level efficiency, the coefficient of technicians and trainees was negative. One reason is that profit-driven systems motivate providers to focus on high-tech care excessively, which do not provide the best health gains for patients. We believe that improvement is based on properly controlled medical tests and channeled change. Although the large number of trainee health workers also leads to inefficiency in medical institutions, under the outbreak’s dynamic, we were still committed to fostering new HRH.

Considering the above analysis, it is clear that the role of pharmacists merits further discussion. Many studies that described the role of the pharmacists during the COVID-19 pandemic reported actions taken by pharmacists, mainly drug information and patient counseling [73]. With the improvement of health care awareness, there is asymmetry between the demand for drug therapy and the lack of clinical pharmacists. Rational use of medicine is among the major health sector problems in most of the countries, but the role of pharmacists as health care professionals is not deemed important by the medical institutions. Azhar studied the pharmacists in Pakistan, and noted that the services of pharmacists were focused towards management more than towards customers [74]. In addition, Chinese clinical pharmacists do not have prescription rights and doctors are typically valued more highly than pharmacists, which often leads to unreasonable drug use. Similar conditions are to be found in other countries. Pharmacists in Malaysia are not the only professionals with the legal right of dispensing medications, and doctors dispense medications as a part of their professional practice [75] as well. The profession is still under continuous transition. With change in the health demands, pharmacists have a further role to play in patient care. There should be an increased focus on professional training of clinical pharmacists and promoting the implementation of prescription rights for pharmacists so that they can provide convenient medical services, reduce irrational drug use, and monitor patients who need long-term medication.

Over the past decade, the Chinese government has increased their investment of medical resources and the number of HRH has increased from 4.9 million in 2007 to 10.2 million in 2019. However, after an increase in the total number of personnel, the proportion of doctors and pharmacists have declined from 43.2 and 6.6% in 2007 to 38.1 and 4.8% in 2019, the main reason is that the proportion of nurses has soared from 31.7% in 2007 to 43.8% in 2019, this helps doctors perform diagnosis and treatment and undoubtedly improve standards of care. According to the *China Health Statistical Yearbook*, China had 3.87 million doctors, 4.45 million nurses and 0.48 million pharmacists in medical institutions in 2019, which accounted for 86.6% of the total number of HRH. Since the abolition of drug price additions, hospital pharmacy windows are full of people and pharmacists spend a lot of time dispensing and supplying medications instead of conducting clinical pharmacy work in hospitals. Whereas previously pharmacists worldwide were seen as responsible primarily for manufacturing and supplying medicines, today the pharmacist’s role has evolved towards a clinical orientation. For massive drug overdoses, there is a general recognition of the urgent need for training more clinical pharmacist and carrying out pharmaceutical care.

## Conclusions and policy recommendations

### Conclusions

This paper combined data envelopment analysis (DEA) with Tobit regression analysis to evaluate the efficiency of health care services in China. The efficiency of health care services was first estimated by using DEA. From the static analysis of medical services efficiency for the year 2007 and 2019, the provinces like Tianjin, Hainan and Tibet were on the frontier and thus considered as efficient provinces in both the two periods. Beijing, Qinghai and Ningxia achieved maximum efficiency only in 2019. The mean overall efficiency was increasing over time, but still far away from the production possibility frontier. The pure technical efficiency was increasing from 0.946 in 2007 to 0.955 in 2019, indicating more efficient than overall efficiency and scale efficiency. From the aspect of dynamic analysis of medical services efficiency at the provincial level, the mean technical efficiency value indicates a 0.7% increase. The mean technological change value of 0.932 indicates an average annual decrease of 6.8% in technological advances, which plays a major role in reducing total factor productivity change (tfpch). The total factor productivity change (tfpch) has shown a similar productivity decline during the study period. In terms of medical services efficiency at the national level, the overall efficiency varied in a way similar to the scale efficiency, showing a trend of decreasing. The pure technical efficiency was higher than overall efficiency and scale efficiency. It appeared to fluctuate from 2011 to 2016, which is influenced by the healthy and medical departments management and technologies. Perhaps part of the reason lies with healthy public policies adjustment.

Tobit regression model was then employed to considered the impact of HRH on the health care services efficiency from the provincial and national level respectively. Based on our above conclusions, results proved that HRH had a generally significant impact on the efficiency. More importantly, pharmacists showed the greatest influence on both the provincial and national health care services efficiency. In the provincial level, the technicians were insignificant, and the correlation coefficient of nurses was negative in the overall efficiency (crste) and scale efficiency (scale) model. In addition, a trend of decreasing returns to scale was showed in most of the provinces, which highlighted that higher proportion of nurses was clearly associated with lower scale efficiency. In the national level, the the correlation coefficient of technicians and trainees was negative. The reason is that profit-driven systems motivate providers to focus on high-tech care excessively, which do not provide the best health gains for patients, and the large number of trainee health workers also leads to inefficiency in medical institutions.

Bridging the inequality of HRH distribution among provinces is a key step to to improve the health system efficiency. This paper used the Theil index to measure inequality trends of HRH distribution. The most severe inequality was pharmacists, and the inequality distribution of doctors and nurses remarkably increased, while that of technicians and trainees was the opposite. If the inequity of HRH had the impact on the health care services efficiency, the pharmacists had greater impact on the efficiency, and one of the important reasons was probably for the highest Theil index. Thus, findings from this study emphasize the need to take more measures to reduce pharmacists quality differences among provinces so as to balance and coordinate medical resources. This will increase the access of medical services and improve health outcomes.

### Policy recommendations

Optimizing the allocation of HRH is a top priority for healthcare systems, the government should adopt a long-term perspective. First, the health sector should pay more attention to increasing the number and quality of HRH in order to narrow the gap across provinces, and establishing policies to attract more health workers to dedicate their life in primary hospitals or rural areas. Secondly, pharmacists have been deemed the health professional nearest to residents. There should be an increased focus on professional training of clinical pharmacists and recognizing the opportunities for pharmacists to improve patient care and providing convenient and efficient medical care for everyday diseases. Finally, more research should be carried out on the layout of HRH, such as discerning the most effective proportion between different types of HRH or allocating HRH among different medical institutions so that we can overcome the bottle necks and achieve national and global health goals in the future.

## Limitations

Previous studies have included the number of beds, financing for health services and other health facility resources to control for potential confounders, effect modification and mediation of these factors. However, in this study, HRH is the only independent variable and no confounders have been adjusted for. This is a key shortcoming in the interpretation of the effect of HRH on the efficiency of medical services in China.

## Data Availability

The datasets used and/or analysed during the current study available from the corresponding author on reasonable request. The data were acquired from the *China Health Statistical Yearbook* (2008–2020), which was published in the Chinese language by the Chinese central government. Please contact author for data requests.

## Abbreviations

HRH: Human resources for health
WHO: World Health Organization
DEA: Data envelopment analysis
OECD: Organization for Economic Co-operation and Development
DMUs: Decision making units
CCR: Charnes, Cooper, and Rhodes
BCC: Banker, Charnes, and Cooper, 1984
CRS: Constant returns to scale
VRS: Variable return to scale
TFP: Total factor productivity change
TECHCH: Technical change
EFFCH: Technical efficiency change
PECH: Pure technical efficiency change
SECH: Scale efficiency change
